# Bacteria in the Adventitia of Cardiovascular Disease Patients with and without Rheumatoid Arthritis

**DOI:** 10.1371/journal.pone.0098627

**Published:** 2014-05-29

**Authors:** Samuel A. Curran, Ivana Hollan, Clett Erridge, David F. Lappin, Colin A. Murray, Gunnar Sturfelt, Knut Mikkelsen, Oystein T. Førre, Sven M. Almdahl, Magne K. Fagerhol, Carl S. Goodyear, Marcello P. Riggio

**Affiliations:** 1 Dental School, University of Glasgow, Glasgow, United Kingdom; 2 Hospital for Rheumatic Diseases, Lillehammer, Norway, and Feiring Heart Clinic, Feiring, Norway; 3 Department of Cardiovascular Sciences, University of Leicester, Glenfield General Hospital, Leicester, United Kingdom; 4 Department of Rheumatology, Lund University Hospital, Lund, Sweden; 5 Hospital for Rheumatic Diseases, Lillehammer, Norway; 6 Department of Rheumatology, Oslo University Hospital, Rikshospitalet, Oslo, Norway; 7 Department of Surgery, University Hospital of North Norway, Tromsø, Norway; 8 Department of Immunology and Transfusion Medicine, Ullevaal University Hospital, Oslo, Norway; 9 Institute of Infection Immunity and Inflammation, University of Glasgow, Glasgow, United Kingdom; Institute of Immunology, Rikshospitalet, Norway

## Abstract

The incidence of atherosclerosis is significantly increased in rheumatoid arthritis (RA). Infection is one factor that may be involved in the pathogenesis of both diseases. The cause of RA and atherosclerosis is unknown, and infection is one of the factors that may be involved in the pathogenesis of both diseases. The aims of this study were to identify bacteria in the aortic adventitia of patients with cardiovascular disease (CVD) in the presence and absence of RA, and to determine the effect of identified candidate pathogens on Toll-like receptor (TLR)-dependent signalling and the proinflammatory response. The aortic adventitia of 11 CVD patients with RA (RA+CVD) and 11 CVD patients without RA (CVD) were collected during coronary artery bypass graft surgery. Bacteria were detected in four samples from CVD patients and three samples from RA+CVD patients and identified by 16S rRNA gene sequencing. *Methylobacterium oryzae* was identified in all three RA+CVD samples, representing 44.1% of the bacterial flora. The effect of *M. oryzae* on TLR-dependent signalling was determined by transfection of HEK-293 cells. Although mild TLR2 signalling was observed, TLR4 was insensitive to *M. oryzae*. Human primary macrophages were infected with *M. oryzae*, and a TLDA qPCR array targeting 90 genes involved in inflammation and immune regulation was used to profile the transcriptional response. A significant proinflammatory response was observed, with many of the up-regulated genes encoding proinflammatory cytokines (IL-1α, IL-1β, IL-6, TNF-α) and chemokines (CCR7, IL-8). The aortic adventitia of CVD patients contains a wide range of bacterial species, and the bacterial flora is significantly less diverse in RA+CVD than CVD patients. *M. oryzae* may stimulate an proinflammatory response that may aggravate and perpetuate the pathological processes underlying atherosclerosis in RA patients.

## Introduction

Premature cardiovascular disease (CVD), as a result of atherosclerosis, is the leading cause of mortality among rheumatoid arthritis (RA) patients [1]. The incidence of CVD is reported to be up to four-fold greater in RA patients, compared to age- and sex-matched controls [1]–[2]. Several traditional risk factors are common to both CVD and RA e.g. obesity, smoking, dyslipidaemia [3], however these cannot fully account for the increased CVD burden observed in RA [2], suggesting that alternative mechanisms are at least partly responsible.

Atherosclerosis and RA are both complex conditions with a strikingly similar autoimmune and inflammatory pathophysiology [4]. Elevated levels of activated T cells and B cells have been observed in both the RA synovium and the atherosclerotic lesion. The persistent ‘high-grade’ systemic inflammation manifest in RA has been suggested to contribute to the elevated atherosclerotic burden [3], [5]. In RA, inflammatory markers are principally expressed in the synovial tissue. Consequently, a variety of over-expressed cytokines such as tumour necrosis factor-α (TNF-α), interleukin-6 (IL-6) and IL-1β, may enter the systemic circulation and alter numerous pathways that potentiate the onset of atherosclerosis [6].

Infection as a contributing factor to both atherosclerosis and RA has received much attention. To date, most studies have concentrated on *Chlamydia pneumoniae* as a possible aetiological agent in atherosclerosis [7]. However, several periodontal bacteria (*Porphyromonas gingivalis*, *Treponema denticola*, *Prevotella intermedia* and *Tannerella forsythia*) have also been isolated from atherosclerotic plaques [8], [9]. Nevertheless, the potential aetiological roles of such bacteria are unknown. Furthermore, data from longitudinal cohort studies have demonstrated that patients with RA are at increased risk of developing infections compared to non-RA subjects [10], and that higher disease activity is associated with a higher probability of developing infections [11]. Moreover, elevated levels of calprotectin (a peptide with antimicrobial and pro-apoptotic effects that is predominantly produced by neutrophils) has been observed in RA serum [12] and atherosclerotic lesions [13]. Additionally, plasma levels of calprotectin in RA have been demonstrated to positively correlate with erythrocyte sedimentation rate, C-reactive protein (CRP) and IgM rheumatoid factor [14].

Accumulating evidence suggests that alterations to the host microbiome precedes RA and may play a key role in pathogenesis [15], however it is equally plausible that alteration to the microbiome follows RA and may lead to vascular co-morbidity. Intriguingly, RA patients exhibit elevated levels of T_H_17 cells (and their signature cytokine, IL-17) in the serum and although normal levels of circulating regulatory T-cells are expressed, they exhibit decreased functionality *ex vivo* [16]. Conceivably, altered T cell functionality in parallel with the elevation of systemic pro-inflammatory mediators characteristic to RA supports a hypothesis of RA-driven dysbiosis and vulnerability to infection by a few select organisms.

A growing body of research supports a novel ‘outside-in’ hypothesis in which vascular inflammation is initiated in the aortic adventitia and progresses inwards to the luminal surface [17]. Furthermore, the aortic adventitia is an area of considerable immunological interest, exhibiting large areas of diffuse inflammatory infiltrate [18]. However, studies carried out to date have not identified potential immunological triggers. In the current study, we explore the hypothesis that the aortic adventitia contains potentially pro-atherosclerotic bacteria and that the microbiome is altered in patients with RA.

## Materials and Methods

### Subjects

The study was undertaken with prior approval of the Regional Ethics Committee for Medical Research (Feiring, Norway), and all patients gave written informed consent before participating. None of the patients were on antibiotic therapy prior to recruitment to the study, but were administered 2 g cefalotin intravenously 15 minutes before surgery.

Aortic samples were collected during coronary artery bypass graft (CABG) surgery of patients with RA (RA+CVD) and without RA (CVD). Study cohorts were enrolled at Feiring Heart Clinic, Feiring, Norway and were age- and gender-matched. All patients were screened for the presence of RA and were evaluated pre-operatively by a single rheumatologist to verify the rheumatologic diagnosis and to assess disease severity and activity. Inclusion criteria for the RA+CVD group were age older than 18 years, a confirmed diagnosis of RA according to accepted criteria [19] and absence of clinically significant malignancy and infection. Inclusion criteria for the CVD group were age older than 18 years and absence of inflammatory rheumatic disease, psoriasis and clinically significant malignancy and infection [18].

### Collection of aortic biopsies

Part of the adventitia covered by the epicardium, from the ventral part of the ascending aorta in connection with the establishment of the proximal aortocoronary anastomoses, was collected as described previously [18]. To avoid thromboembolic complications, the anastomoses were established in areas with less pronounced gross signs of atherosclerosis. Biopsies were snap frozen at time of surgery and stored at −80°C prior to analysis.

### DNA extraction from tissue samples

At all stages strict aseptic techniques were used. Tissue samples were homogenised on dry ice in 1 mL TRIzol (Invitrogen, Paisley, United Kingdom) using a TissueRuptor kit and sterile probe (Qiagen, Crawley, United Kingdom). 200 µL of chloroform was added, followed by incubation at room temperature for 15 minutes then centrifugation at 12x*g* for 15 minutes at 4°C. The aqueous phase was removed and the remaining interphase and organic phases were suspended in 300 µL of 100% ethanol and the sample centrifuged at 12x*g* for 5 minutes to pellet the DNA. The pellet was washed twice in 1 mL 100 mM sodium citrate before re-suspension in 1 mL 75% ethanol. This was then centrifuged at 12x*g* for 5 minutes at room temperature and the supernatant removed. The remaining pellet was dissolved in 100 µL of sterile water, and the DNA containing supernatant removed and stored at −20°C. Control samples containing sterile water instead of tissue were run in parallel to monitor for sterility of reagents and apparatus.

### PCR amplification

PCR amplification of the 16S rRNA gene was performed using the universal primer pair 5′-CAGGCCTAACACATGCAAGTC-3′ (63f) and 5′-GGGCGGWGTGTACAAGGC-3′ (1387r), which amplified a 1325 bp segment of the 16S rRNA gene. PCR reactions were performed in a total volume of 50 µL containing 5 µL of the extracted DNA and 45 µL of reaction mixture comprising 1x GoTaq PCR buffer (Promega, Southampton, United Kingdom) 1.25 units GoTaq polymerase (Promega, Southampton, United Kingdom), 1.5 mM MgCl_2_, 0.2 mM dNTPs (New England Biolabs, Hitchin, United Kingdom), and each primer at a concentration of 0.2 µM. Thermal cycling comprised one cycle of 95°C for 2 minutes, followed by 35 cycles of 95°C for 1 minutes, 60°C for 1 minutes and 72°C for 1.5 minutes, followed by a final extension cycle at 72°C for 10 minutes.

### PCR quality control

When performing PCR, stringent procedures were employed to prevent contamination. Negative and positive controls were included with each batch of samples being analysed. The positive control comprised a standard PCR reaction mixture containing 10 ng of *Escherichia coli* genomic DNA instead of sample, whereas the negative control contained sterile water instead of sample. Each PCR product (10 µL) was subjected to electrophoresis on a 2% agarose gel, and amplified DNA was detected by staining with ethidium bromide (0.5 µg/mL) and examination under ultraviolet illumination.

### Cloning of PCR products

PCR products were cloned into the StrataClone PCR cloning vector pSC-A-amp/kan (Agilent Technologies, Wokingham, United Kingdom) in accordance with the manufacturer's instructions.

### PCR amplification of 16S rRNA gene inserts

Following cloning of the 16S rRNA gene products amplified by PCR for each sample, approximately 50 clones from each generated library were randomly selected. The 16S rRNA gene insert from each clone was amplified by PCR with the primer pair 5′-TGTAAAACGACGGCCAGT-3′ (M13 forward) and 5′- GAGCGGATAACAATTTCACACAGG - 3′ (M13 reverse).

### Restriction enzyme analysis

Each re-amplified 16S rRNA gene insert was subjected to restriction enzyme analysis. 0.5 µg of each PCR product was digested in a total volume of 20 µL with 2.0 units of each of the restriction enzymes *Rsa*I and *Mnl*I (Fermentas Life Sciences, York, United Kingdom) at 37°C for 2 hours. Restriction fragments were visualised by agarose gel electrophoresis. For each library, clones were initially segregated into groups based upon their *Rsa*I restriction digestion profiles. Further discrimination was achieved by digestion of clones with *Mnl*I, and clones with identical restriction profiles for both enzymes were finally grouped together in distinct restriction fragment length polymorphism (RFLP) groups.

### DNA sequencing

16S rRNA gene PCR products insert of a single representative clone from each RFLP group were sequenced. Sequencing was performed using the SequiTherm EXCEL II DNA Sequencing Kit (Cambio, Cambridge, United Kingdom) and IRD800-labelled 357f sequencing primer (5′-CTCCTACGGGAGGCAGCAG-3′). The following cycling parameters were used: (i) initial denaturation at 95°C for 30 seconds; (ii) 10 seconds at 95°C and 30 seconds at 70°C for 15 cycles. Formamide loading dye (6 µL) was added to each reaction mixture after thermal cycling and 1.5 µL of each reaction mixture was run on a LI-COR Gene ReadIR 4200S automated DNA sequencing system.

### DNA sequence analysis

Sequencing data were converted to FASTA format and compared with bacterial 16S rRNA gene sequences from the EMBL and GenBank sequence databases using the BLAST program [20]. The program was run through the National Centre for Biotechnology Information website (http://www.ncbi.nlm.nih.gov/BLAST). Clone sequences that demonstrated at least 98% identity with a known sequence from the database were considered to be the same species as the matching sequence with the highest score. Clone sequences possessing less than 98% identity were classified as potentially novel phylotypes.

16S rRNA clone sequences have been deposited in GenBank with the following accession numbers:

BankIt1722590 SEQ1 KJ767741

BankIt1722590 SEQ2 KJ767742

BankIt1722590 SEQ3 KJ767743

BankIt1722590 SEQ4 KJ767744

BankIt1722590 SEQ5 KJ767745

BankIt1722590 SEQ6 KJ767746

BankIt1722590 SEQ7 KJ767747

BankIt1722590 SEQ8 KJ767748

BankIt1722590 SEQ9 KJ767749

BankIt1722591 SEQ1 KJ767750

BankIt1722591 SEQ2 KJ767751

BankIt1722591 SEQ3 KJ767752

BankIt1722591 SEQ4 KJ767753

BankIt1722591 SEQ5 KJ767754

BankIt1722591 SEQ6 KJ767755

BankIt1722591 SEQ7 KJ767756

BankIt1722591 SEQ8 KJ767757

BankIt1722591 SEQ9 KJ767758

BankIt1722591 SEQ10 KJ767759

BankIt1722591 SEQ11 KJ767760

BankIt1722591 SEQ12 KJ767761

BankIt1722591 SEQ13 KJ767762

BankIt1722591 SEQ14 KJ767763

BankIt1722591 SEQ15 KJ767764

BankIt1722591 SEQ16 KJ767765

BankIt1722591 SEQ17 KJ767766

BankIt1722591 SEQ18 KJ767767

BankIt1722591 SEQ19 KJ767768

BankIt1722591 SEQ20 KJ767769

BankIt1722591 SEQ21 KJ767770

BankIt1722591 SEQ22 KJ767771

BankIt1722591 SEQ23 KJ767772

BankIt1722591 SEQ24 KJ767773

BankIt1722591 SEQ25 KJ767774

BankIt1722591 SEQ26 KJ767775

BankIt1722591 SEQ27 KJ767776

BankIt1722591 SEQ28 KJ767777

Calprotectin immunohistochemistry.

Slides were prepared as previously described [18]. Briefly, citrate heat antigen retrieval was performed followed by blocking with 2.5% normal horse serum. Tissue sections were then incubated with monoclonal rabbit anti-human calprotectin primary antibody, or an isotype control, overnight at 4°C, followed by incubation with 0.5% hydrogen peroxide. Secondary HRP-conjugate secondary antibodies were incubated for 1 hour. Binding was visualised with ImmPACT DAB peroxidase substrate (Vector Labs, Peterborough, United Kingdom). Slides were counterstained with hematoxylin. The main negative control used was an isotype control antibody plus the secondary antibody.

### Culture of Methylobacterium oryzae

*Methylobacterium oryzae* 18207^T^ was obtained from the DSMZ culture collection (DSMZ, Braunschweig, Germany) and cultured at 30°C in nutrient agar (0.5% peptone, 0.3% meat extract, 1.5% agar, pH 7.0).

### Isolation and culture of CD14+ monocytes

CD14+ monocytes were isolated from human buffy coats using the EasySep positive selection kit (StemCell Technologies, Grenoble, France) in accordance with the manufacturer's instructions. Isolated cells were tested for purity by FACS analysis. CD14+ monocytes were then cultured for seven days at 37°C in 5% CO2 in RPMI-1640 medium (Sigma-Aldrich, Poole, United Kingdom) supplemented with 200 mM L-glutamine (Life Technologies, Paisley, United Kingdom), 10000 units Pen-strep (Life Technologies, Paisley, United Kingdom) and 10% foetal calf serum (Sigma-Aldrich, Poole, United Kingdom). CD14+ monocytes were differentiated into macrophages in the presence of 15 ng/mL human M-CSF (Peprotech, London, United Kingdom).

### Primary macrophage challenge with *M. oryzae*, RNA isolation and cDNA synthesis

Macrophages were cultured until 70% confluence was achieved and then challenged with exponentially growing *M. oryzae* (multiplicity of infection 200) or vehicle control. Cells were harvested at four and eight hours post-infection and total cellular RNA purified using the RNeasy Kit (Qiagen, Crawley, United Kingdom). cDNA was prepared by reverse transcription of RNA (500 ng) using a High Capacity RNA-to-cDNA Kit (Life Technologies, Paisley, United Kingdom).

### TaqMan mRNA analysis

Transcriptional activity of primary human macrophages following infection with *M. oryzae* was determined using Human Immune TaqMan Low Density Arrays (Life Technologies, Paisley, United Kingdom) in accordance with the manufacturer's instructions. 400 ng of DNase-treated cDNA was used in each sample. Gene expression was quantified using the comparative threshold (C_T_) method with GAPDH as the endogenous control.

### TLR transfection assay

HEK-293 cells were plated in 96-well plates at 8×10^3^ cells per well and transfected after 24 hours using GeneJuice (Novagen, Watford, United Kingdom) with 30 ng of human TLR2, TLR4 (co-expressing MD-2), 30 ng pCD14, 20 ng renilla luciferase-reporter construct (pRL-TK) and 10 ng firefly luciferase-reporter construct driven by the NF*k*B dependent E-selectin promoter (pELAM) cloned into pGL3 (Promega, Southampton, United Kingdom). Cells were grown for two to three days post-transfection prior to 18-hour challenge. Reporter levels were normalised to co-transfected renilla luciferase and represented as fold induction relative to cells cultured in medium alone.

## Results

### Patient characteristics

The characteristics of each study population are shown in **Table 1**. The RA+CVD group had increased levels of inflammatory biomarkers i.e., CRP, history of myocardial infarction and use of oral steroids, Cox2 inhibitors and disease-modifying anti-rheumatic drugs. However, use of other medication was similar between the groups. Conversely, patients without RA (CVD group) had an increased familial history of CVD.

**Table 1:** Patient characteristics.

Characteristics	16S rRNA gene sequencing		Histology	
	CVD (n = 11)	RA+CVD (n = 11)	CVD (n = 20)	RA+CVD (n = 19)
Age, years	68±8	66±9	68±9	69±9
Males (%)	4 (36)	4 (36)	14 (70)	12 (63)
Current smokers (previous smokers)	0 (6)	2 (5)	1 (12)	2 (11)
CRP, mg/L	3.3±2	10±9	3±3	23±40
Duration of coronary artery disease, years	5.1±4	2.6±4	7.2±6.6	6.3±8.7
Time from angiography to CABG, days	6.7±8	9.7±8	31±60	17±30
Acute coronary syndrome (%)	1 (9)	4 (36)	4 (20)	9 (47)
Hypertension (%)	8 (72)	4 (36)	9 (45)	13 (68)

Demographic and clinical characteristics of patient from whom samples were analysed by histology and 16S rRNA gene sequencing. Where applicable, values represent mean ± SD.

### Evaluation of calprotectin in the aortic adventitia

**Figure 1:**
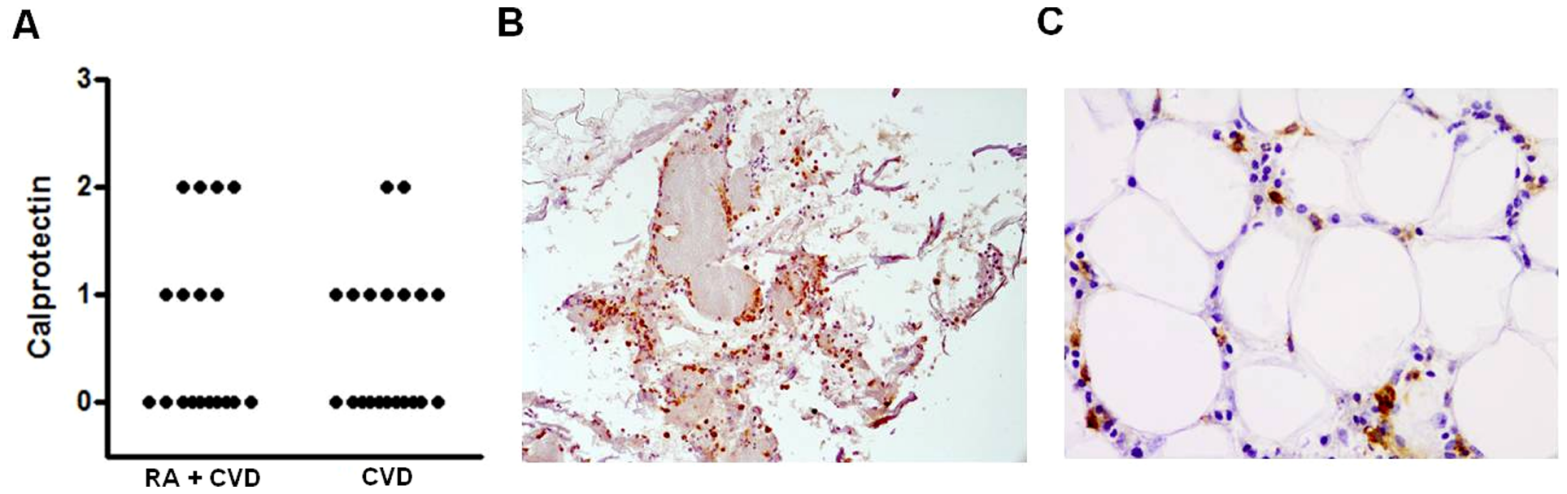
Calprotectin presence in the inflammatory infiltrate of the aortic adventitia of late stage CVD patients. (A) Immuno-histological quantification of calprotectin in the aortic adventitia of RA+CVD (n = 19) or CVD (n = 20) patients. The number of inflammatory cells was counted and each section was scored according to the percentage of cells expressing calprotectin. 0, negative; 1, >0%<10%; 2, 10–25%; 3, >25%. Representative images show calprotectin staining within the inflammatory infiltrate at (B) 10x and (C) 40x magnifications.

Detection of calprotectin was conducted on 19 RA+CVD samples and 20 CVD samples. Immunohistochemical staining demonstrated that calprotectin was expressed in areas of mononuclear cell infiltration and was detectable in 8 of the 19 RA+CVD patients (42.1%) and 9 of the 20 CVD patients (45%) (**Figure 1**). There was no significant difference in either the number of patients expressing calprotectin or the intensity of calprotectin expressed across the two cohorts.

### Molecular detection of bacteria in the aortic adventitia

**Figure 2:**
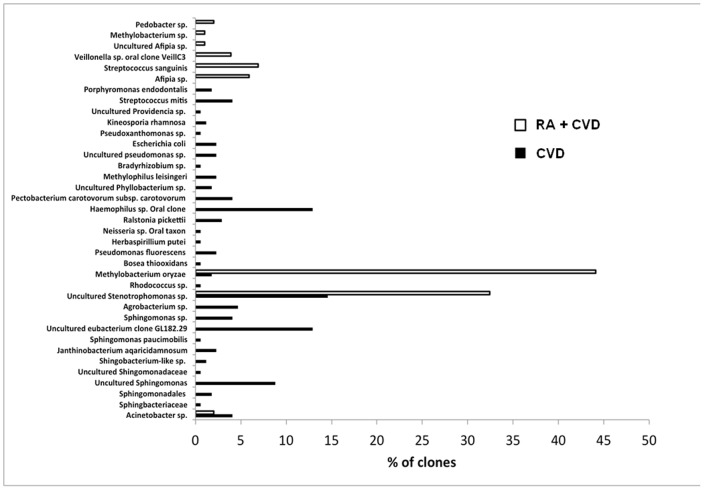
Bacterial species identified by 16S rRNA gene sequencing. Overall prevalence of bacterial species identified in all samples from the RA+CVD and CVD cohorts is shown. Bars depict the overall % of clones representing each bacterial species from the aortic adventitia of three RA+CVD patients (white bars) and four CVD patients (black bars).

16S PCR confirmed that 4 of 11 CVD and 3 of 11 RA+CVD patients had detectable levels of bacteria present in the aortic adventitia. A total of 273 clones from the seven 16S rRNA PCR-positive samples were subjected to RFLP analysis and a single representative clone from each RFLP group was sequenced. Subsequent NCBI BLAST gene alignments revealed a broad range of bacterial species. The bacteria identified in each of the 3 RA+CVD and 4 CVD 16S rRNA PCR-positive samples and their relative prevalence in each sample is shown in **Figure 2**.

The four samples from CVD patients contained 31 different bacterial phylotypes and the three positive samples from RA+CVD patients contained nine different bacterial phylotypes. This difference in the heterogeneity of bacteria between RA+CVD and CVD patients was statistically significant (*p*<0.00001).

In the CVD group the predominant species were uncultured *Stenotrophomonas* sp. (14.6% of clones analysed), uncultured eubacterium clone (12.9%) and *Haemophilus* sp. oral clone (12.9%). In the RA+CVD group the predominant species were *Methylobacterium oryzae* (44.1%), which was present in all three 16S rRNA PCR-positive RA+CVD samples, and uncultured *Stenotrophomonas* sp. (32.4%).

### Effect of *M. oryzae* on TLR-dependent signaling

**Figure 3:**
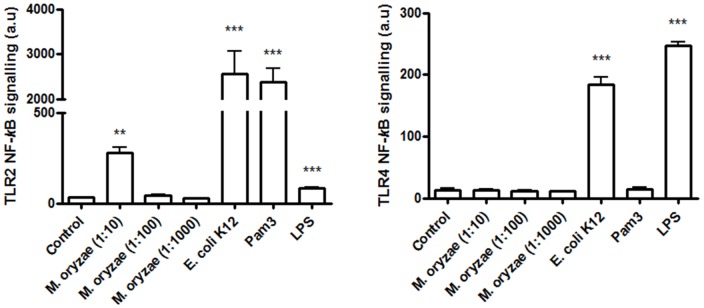
Effect of *M. oryzae* on TLR-dependent signaling. HEK-293 cells co-transfected with NFkB sensitive reporter (pELAM) and TLR2 and TLR4 were challenged for 18 hours with *M. oryzae* (1∶10 dilution equivalent to 1×10^7^ cells/mL), *E. coli* K12 (1×10^7^ cells/mL), pam3CSK4 (10 ng/mL) and LPS (10 ng/mL). The vehicle control was treated with medium only. Arbitrary units (a.u.) represents mean fluorescent intensity of the reporter construct normalised to co-transfected renilla. Data are representative of three experiments ± S.D. ^**^ *p*<0.01 vs. control; ^***^ *p*<0.001 vs. control.

While whole *E. coli* and purified lipopolysaccharide (LPS) were able to stimulate nuclear factor kappa B (NFκB) signalling via TLR4 in transfected HEK-293 cells, whole *M. oryzae* failed to do so (**Figure 3**). However, *M. oryzae* was able to stimulate TLR2-mediated NFκB signalling albeit at a lower level than Pam3SK4 or whole *E. coli*. Subsequent hot phenol extraction followed by sodium dodecyl sulphate polyacrylamide gel electrophoresis confirmed the absence of the TLR4 ligands LPS and lipopolyoligosaccharide (LOS) in *M. oryzae* (data not shown).

### Proinflammatory responses to *M. oryzae* infection in human primary macrophages

**Figure 4:**
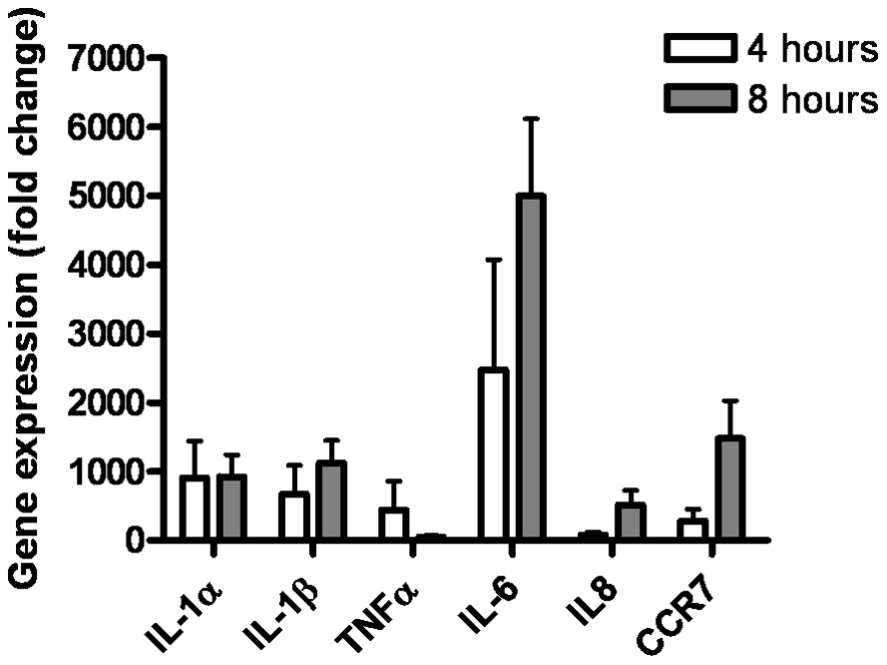
Response of human primary macrophages following *M. oryzae* infection. Primary macrophages were infected with exponentially growing *M. oryzae* (MOI 200) for four and eight hours. Each column represents the mean ± standard error of three experiments.

We have evidence that the human aortic adventitia possesses significant numbers of CD68^+^ macrophages (Carl Goodyear, unpublished data). Although, macrophages express both TLR2 and TLR4 on their cell surface, a range of other non-opsonic pattern recognition receptors also support their recognition of pathogens. To further characterise the potential atherogenic role of *M. oryzae* we infected human primary macrophages and determined the relative expression of 90 genes involved in inflammation and immune regulation. Following four and eight hours of infection with *M. oryzae*, macrophages displayed an excessive pro-inflammatory response. After both 4 and 8 hours of infection, many of the up-regulated genes were those that encode pro-inflammatory cytokines (IL-1α, IL-1β, IL-6 and TNF-α) and chemokines (CCR7 and IL-8) (**Figure 4**), all of which have been implicated in RA and CVD pathogenesis. This inflammatory milieu may aggravate and perpetuate the pathological processes underlying atherosclerosis in RA patients.

## Discussion

This novel study demonstrated the presence of bacterial DNA in aortic adventitia of patients with CVD. A mixed bacterial population was detected in the aortic adventitia in both RA+CVD and CVD patients, but it was less heterogeneous in the RA+CVD group. In RA+CVD, *M. oryzae* appeared in all samples positive for bacterial DNA presence. Further investigation confirmed that this bacterial species triggers a robust pro-inflammatory response by macrophages following infection. Taken together, these data suggest that the aortic adventitia environment is susceptible to microbial colonisation and that RA pathophysiology selects for specific bacterial species, or *vice versa*.

Atherosclerosis pathogenesis has been independently suggested to originate from both the intima and the adventitia, but is now recognised as a disease of the entire vessel [17]. However, an understanding of how the adventitial microenvironment functions in atherosclerosis pathology and how co-morbidities such as RA influence this site has received little attention.

In the current study we demonstrate for the first time that the anti-microbial peptide calprotectin, an inflammatory biomarker released from neutrophils, is expressed within the human aortic adventitia of CVD patients with and without RA. Calprotectin is involved in inflammation and in the response to infection. Calprotectin-expressing monocytes identified in the ascending aorta of ApoE^−/−^ mice have been suggested to regulate monocyte transmigration and endothelial activation [21]. Importantly, calprotectin expression resided in areas of diffuse inflammation within the adventitia. A number of potential triggers, including hypertension, lipoproteins, smoking and oxidative stress, are capable of inducing a vascular pro-inflammatory response, However, an interesting hypothesis is that infectious agents may play a role in atherosclerosis, either through a direct pro-inflammatory role on the vessel wall or through a less specific, systemic pro-inflammatory effect [22]. In theory, calprotectin may play a role in antimicrobial defence in the vessel wall.

The ability of a diverse range of bacterial species to infect atherosclerotic tissue has been well documented [8], [23], [24]. However, it has been proposed that atherosclerotic tissue may act as a ‘mechanical sieve’ trapping bacteria present in blood circulation and that detected bacteria, although present, may have no pathological significance [24]. In contrast, our study was confined to the adventitia and was from areas of minimal macroscopic signs of atherosclerosis.

The current study supports the hypothesis that bacterial infection may contribute to the pathophysiology of atherosclerosis. The bacteria identified included an array of environmental and oral species. Most of these species have previously been reported as opportunistic or nosocomial pathogens. For example, members of the *Stenotrophomonas* genus, which were detected in both RA+CVD and CVD patients, have recently emerged as important opportunistic pathogens in debilitated individuals [25], and have been reported to infect immunocompromised individuals with increasing frequency [26]. *Stenotrophomonas maltophilia* has been demonstrated to cause blood-stream infections [27]. Furthermore, several species of *Stenotrophomonas* including *S. maltophilia*, express a protease (*StmPr1*) capable of breaking down fibrinogen, fibronectin and collagen [28], which could cause local tissue damage and as such may be a potential atherogenic trigger.

*M. oryzae* was detected in the adventitia of all three 16S rRNA gene-positive RA+CVD patients, and may act as a primary pathogen. However, eight samples did not appear to harbour bacterial DNA (as evidenced by a negative 16S rRNA PCR result). This could be due to bacteria being present at a level below that detectable by the standard PCR detection method used. It could be also be possible that bacteria contributed to disease pathology at a time prior to sampling. The occurrence of *M. oryzae* in vessels may occur in a patchy pattern and differs in segments of the vascular tree, thus it is possible that we did not detect all cases with *M*. *oryzae* in the aorta since we examined only a small aortic specimen and not the whole aorta. Alternatively, the pathology in these eight non-infected samples could be driven by other unknown mechanisms. Interestingly, *Methylobacterium* sp. has previously been identified in human aortic aneurysm samples [29]. Although knowledge of *M. oryzae* is lacking, insight may be gained from considering its closest known relative, *M. mesophilicum* [30]. Interestingly, *M. mesophilicum* has been reported as a cause of opportunistic infections in immunocompromised hosts [31] and has been isolated from several clinical sites, including blood, synovial, and cerebral spinal fluid [32]–[34]. Although the source of infection remains unclear, infection has been associated with environmental exposure. In particular, *M. mesophilicum* contamination of hospital tap water has been implicated as the source of an isolated nosocomial outbreak [35].

Previous studies have demonstrated that TLR2 and TLR4 are the most abundant TLRs in human atherosclerotic lesions [36]. TLR2 mediates host responses to bacteria principally by recognition of the lipopeptides (e.g. PAM3), and TLR4 mediates host responses to bacteria by recognition of LPS. In the current study we demonstrated that although *M. oryzae* does not activate TLR4 signalling (due to non-detectable levels of LPS) and stimulates only mild TLR2 signalling, this bacterium can trigger an aggressive macrophage-mediated pro-inflammatory response that may potentiate atherosclerosis.

It has been suggested that RA patients exhibit a distinctive oral enterotype, characterised by the overabundance of a single and virulent *Porphyromonas* species [37]. It can be speculated that a reduction in oral bacterial heterogeneity may reduce the diversity of microbes entering the bloodstream. Furthermore, RA is characterised by a chronic systemic T-cell response [38]–[39] and increased expression of pro-inflammatory mediators and cytokines such as TNF-α, IL-17 and IL-1. This may explain the reduction in bacterial heterogeneity in the adventitia of RA+CVD patients observed in the present study. Conceivably, the increased systemic inflammatory state associated with RA may reduce the ability of certain bacteria to invade the body and enter systemic circulation, although this remains to be established. Also, anti-rheumatic treatment may contribute to an imbalance in the immune system, resulting in the different bacterial populations observed between RA+CVD and CVD patients.

Complement C2-deficient individuals have a high frequency of severe infections and systemic lupus erythematosus-like disorders [40]. Interestingly they also have a very high frequency of CVD, as has been shown for RA patients in our current study.

Although our current study is relatively small, it is still important as no similar studies have been performed earlier and, due to feasibility, studies on surgical vascular specimens in RA are often lacking or small [41].

In conclusion, our study suggests that bacterial colonisation of the aortic adventitia might contribute to the development of atherosclerotic disease. Furthermore, the immunological and pathological alterations that accompany RA may coincide with a reduced bacterial heterogeneity, allowing only a few select organisms to successfully colonise. One such organism may be *M. oryzae*. However, further studies are required to investigate a potential role for this microorganism in atherosclerotic pathology.
